# Sensitivity Analysis of Long Short-Term Memory-Based Neural Network Model for Vehicle Yaw Rate Prediction

**DOI:** 10.3390/s25051363

**Published:** 2025-02-23

**Authors:** János Kontos, László Bódis, Ágnes Vathy-Fogarassy

**Affiliations:** 1 Continental Automotive Hungary Ltd., H-8200 Veszprém, Hungary; kontosjan@gmail.com (J.K.); laszlo.bodis@continental-corporation.com (L.B.); 2 Department of Computer Science and Systems Technology, University of Pannonia, H-8200 Veszprém, Hungary

**Keywords:** long short-term memory network, yaw rate prediction, sensitivity analysis, experimental data

## Abstract

In recent years, the application of artificial neural network models has become increasingly widespread in the automotive industry; however, the sensitivity analysis of these models is often neglected. This shortfall poses significant risks in safety-critical applications, where the reliability of models under varying conditions is of critical importance. This study focuses on the sensitivity analysis of a long short-term memory neural network model, previously published by us, designed to predict the future yaw rates of vehicles. Our research aimed to determine the minimum amount of data required for effective model training and to conduct a comprehensive sensitivity analysis, examining the performance and applicability of the trained model under varying tire pressures, different passenger loads, and different passenger configurations. Additionally, we investigated whether the trained model could be applied to other vehicle types. Our results indicated that the vehicle weight distribution was the most influential factor affecting the accuracy of the model. Nonetheless, the model’s predictive error remained consistently within the safety thresholds defined by the standards under all tested conditions. Our experiments and analyses were performed using over 7.5 h of data collected under real-world conditions, which will be freely available to the research community.

## 1. Introduction

Currently, the automotive industry stands as one of the largest consumers of various black box models, including neural networks. The most prominent application area is autonomous driving. However, there are numerous other highly significant but smaller models utilized for specific tasks within the industry. However, despite their critical role, sensitivity analyses of these models are frequently overlooked, which can lead to significant issues, particularly in applications where safety is paramount. Conducting thorough sensitivity analyses is crucial to ensure the reliability of these models.

In this study, we performed a sensitivity analysis of our previously published neural network model [1]. The model was designed to predict the yaw rate of a vehicle 0.2 s in the future. The yaw rate, defined as the vehicle’s angular velocity around its center of mass and measured in degrees per second (deg/s), is a crucial input for the vehicle’s electronic brake system. A primary recipient of this signal is the electronic stability control (ESC), which plays a crucial role in maintaining the directional stability and control of a vehicle during critical driving maneuvers. Therefore, predicting the yaw rate is a highly important safety-critical task, and conducting a sensitivity analysis of the prediction’s accuracy holds significant practical value. The developed model integrates historical and real-time data, encompassing the four-wheel speed, lateral and longitudinal acceleration, front-axle steering angle, and yaw rate. Considering the limited hardware resources of the brake system, these constraints were also taken into account during model development. The detailed methodology for the establishment of the model is documented in our previous publication [1].

In this article, we focus on the applicability of the previously developed model. When the model is applied in real-life scenarios, several questions arise: How many and which types of data are required to train the model? Can the model be applied under different conditions (e.g., varying tire pressures, differing numbers of passengers), and what is the magnitude of the prediction error for a generally trained model when used under conditions different from those in training? Finally, can a model trained on a specific type of vehicle be applied to another type of vehicle, and, if so, what prediction error can be expected during its application? To address these questions, (1) experimental data were collected to refine the developed method, enhancing its accuracy and reliability, and (2) extensive sensitivity analyses were performed in different driving scenarios. The experimental data collected will be made publicly available to other researchers to support further studies.

The structure of this article is organized as follows. Section 2 reviews the existing literature related to the prediction of the yaw rate. Section 3 introduces the previously fine-tuned long short-term memory (LSTM) architecture, including its parameters and hyperparameters. The subsequent Section 4 introduces the data collection and preprocessing procedure, details the method used to determine the necessary amount of training data, and outlines the methodology for the sensitivity analysis. Section 5 presents the results of the enhanced model, the training data sufficiency assessment, and the sensitivity analysis. In Section 6, a comparison of our results with other models in the literature is provided and we highlight the key conclusions of our analysis. Finally, Section 7 summarizes the paper.

## 2. Literature Review

This section reviews the key literature on the prediction of vehicles’ dynamic parameters, with a focus on the yaw rate and the use of artificial neural networks (ANN) in safety-critical automotive applications.

The yaw rate is a crucial vehicle dynamic parameter, influenced by the road–tire friction [2]. It is measured along with the longitudinal and lateral acceleration using an integrated measurement unit (IMU) near the center of mass of the vehicle [3]. Proper monitoring and verification of the IMU’s mounting position is essential to ensure accurate data acquisition [4]. Research shows latency between the steering input and resultant yaw rate, ranging from 50 ms to 200 ms, depending on the maneuvering and road conditions [5].

Yaw rate prediction plays a crucial role in autonomous driving by enabling precise vehicle state estimation, which is essential for trajectory planning and stability control. Accurate forecasting of the yaw rate allows autonomous systems to make real-time adjustments in steering and braking, enhancing the safety during dynamic maneuvers. Furthermore, yaw rate prediction is vital in trajectory prediction for autonomous driving, as it helps in anticipating the vehicle’s future position and orientation [6]. This capability is particularly important for collision avoidance, lane keeping, and path-following algorithms, ensuring smooth and efficient navigation even in complex driving environments. The integration of artificial neural networks in yaw rate estimation improves the adaptability of self-driving vehicles to changing road conditions, contributing to more reliable and robust autonomous navigation [7].

Vehicle state estimation, including the sideslip angle, vehicle velocity, roll, and yaw rate, is extensively studied, with significant literature and reviews [8,9,10]. The roll and pitch rates, like the yaw rate, relate to different dimensions of vehicle movement, such as tilting during turns and elevation changes during acceleration and braking. There is substantial research on the prediction of these dynamic attributes [11,12]. ANNs are widely used to regulate yaw rates, primarily as control algorithms rather than estimation methods [13,14,15].

The literature on yaw rate prediction can be categorized into three clusters: data-driven estimators like ANNs, dynamic model-based solutions using filtering techniques or observers, and computer vision-based approaches. ANNs are adaptable and can learn from data without precise mathematical models, making them ideal for dynamic or uncertain environments. Kalman filters, however, require accurate system models and well-defined noise statistics but can handle nonlinear behaviors with adaptive versions [16].

Extensive research explores ANN-based strategies in automotive safety mechanisms [17,18]. The yaw rate is critical for various ANN-oriented models in estimating the vehicle state [19,20]. The early and significant contributions of Karri and Butler highlight advances in input parameter selection, network architectures, and optimization for ANN-based yaw angle prediction [21]. Hermansdorfer et al. developed a comprehensive ANN framework for the simulation of vehicle dynamics, using inputs like the velocity, yaw rate, acceleration, steering angle, torque, and braking pressure. Despite its comprehensiveness, it does not predict future states [22].

Dynamic model-based solutions face limitations due to the need for extensive vehicle-specific parameters. Novara et al. introduced a set membership filter design for yaw rate prediction, notable for its direct data identification without an additional model [23]. Sum et al. proposed an estimation framework for autonomous vehicles with an innovative tire model and adaptive estimation using a square-root cubature Kalman Filter, validated through simulations [24]. Moussa et al. explored an Extended Kalman Filter (EKF) for the estimation of the lateral velocity and yaw rate, incorporating a nonlinear tire model for improved precision [25]. Baffet et al. presented a two-block EKF approach for the estimation of dynamic parameters using standard sensors [26]. Zhang et al. developed an Extended H-Infinity Kalman Filter (EH∞KF) for vehicle dynamics estimation, validated through simulations [27]. Shi et al. proposed a linear Kalman filter using the magnetic angular rate and gravity sensors to mitigate magnetic disturbances [28].

Interest in computer vision for yaw rate detection is increasing. This technique can forecast the yaw rate without additional sensors, using existing vehicle cameras. However, it does not inherently predict future yaw rates. Huang et al. introduced the yaw angle estimation network (YAEN), a deep learning framework using a monocular camera for yaw angle estimation, which requires more hardware resources than typical electronic brake systems [29]. Cunliang et al. developed an adaptive neural network control based on You Only Look Once V5 (YOLOv5) for steering angle prediction, trained on real road images. Despite showing a strong correlation between the steering angle and yaw, it requires large volumes of hardware resources [30].

Unfortunately, the existing literature lacks studies that specifically address the sensitivity analysis of dynamic vehicle models, particularly in relation to yaw rate prediction methods. This gap underscores the novelty and significance of our work.

## 3. Predicting Yaw Rate with Long Short-Term Memory Neural Network

The main objective of this study was to prepare a detailed sensitivity analysis for the previously constructed neural network model, capable of confidently forecasting forthcoming values for the vehicle’s yaw rate. The available signals inside the vehicle are highly dependent on the producer of the vehicle. The ECUs built into the vehicle can have various architectures for communication, influencing the availability and accessibility of different sensor signals. Due to these variations, this study focused on the selection of mandatory signals required for the brake system, ensuring that the model remains applicable across different vehicle platforms. The proposed neural network utilizes past and current data on the longitudinal and lateral acceleration ($a_{long}$, $a_{lat}$), yaw rate (Φ), steering angle for the front axis (δ), and wheel speeds (*v*) as its inputs. The longitudinal acceleration represents the rate of change in the vehicle’s speed along the forward/backward axis, while the lateral acceleration is perpendicular to the longitudinal direction. Both accelerations are quantified in g units. The yaw rate is measured in deg/s. Wheel speeds (specifically, FL for the front left, FR for front right, RR for rear right, and RL for rear left) are the most convenient parameters in characterizing vehicle movement and are measured in km/h. The steering angle for the front axis refers to the angle at which the front wheels of a vehicle are turned relative to their straight-ahead position. This angle is measured in degrees and indicates the direction in which the wheels are pointed.

Given its ability to effectively capture and leverage long-term temporal dependencies, which is crucial for time-series prediction, the long short-term memory network emerged as the preferred choice. This decision was further supported by the need to account for the dynamics and inertia inherent in vehicular motion, leading us to pursue the development of an LSTM-based network. A detailed description of the LSTM neuron used can be found in our previous article [1].

**Figure 1:**
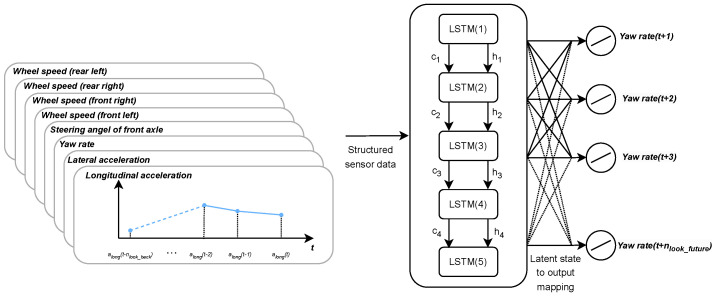
The structure of the developed neural network. Structured sensor data, including wheel speeds, steering angle, yaw rate, and accelerations, serve as inputs. The LSTM layers process these inputs over time, maintaining the cell state ($c_{t}$) and hidden state ($h_{t}$) to capture temporal dependencies. The final latent representations are mapped to the output layer, predicting future yaw rate values at multiple time steps.

To develop an optimal neural network with low computational complexity and high accuracy, an extensive hyperparameter tuning process was undertaken. The hyperparameters of the network were tuned using the hybrid search method described in [31], considering the fact that the model would be integrated into an electric control unit with limited hardware resources. The prediction interval for the LSTM network was set at 20 following timestamps, allowing the network to predict yaw rate values up to 200 ms ahead, according to the literature and safety considerations [5,17]. The experiments determined that the optimal size of the look-back time window was 30 loops, using data from the past 0.3 s to predict the yaw rate 0.2 s into the future. Parameter optimization used the Adam optimizer [32] with a learning rate of 0.001, monitoring the mean squared error as the loss function. Early stopping was applied with a patience value of 5 to prevent overfitting. The optimal batch size for training was determined to be 512. The final model consists of only one hidden layer with 5 LSTM units and does not contain dropout regularization. The input to the network comprises the past and current values of the input variable, while the output predicts the future values of the yaw rate. The details of the hyperparameter tuning are summarized in Table 1, and the structure of the developed model can be seen in Figure 1. This network configuration, along with its parameters and hyperparameters, will be referred to as the *base LSTM network structure*. For more details, see [1].

## 4. Research Methodology

### 4.1. Study Data

To evaluate the applicability of the proposed model, we intentionally avoided using the same dataset that was used during model development. Instead, we gathered a new, more extensive dataset for our research.

#### 4.1.1. Collecting Baseline Data

The data used in the study were collected by qualified drivers during tests conducted at Continental’s test track in Gyulafirátót, Hungary. The same 2022 commercial sport utility vehicle (SUV) (*Vehicle Original*) was employed to collect the baseline data, consistent with its use in our previous study [1]. This vehicle was specifically selected for its distinctive features, such as a high center of mass, considerable vehicle mass, and advanced suspension systems, all of which are critical factors in accurately capturing the necessary dynamic behaviors. An internal measurement system recorded the longitudinal and lateral acceleration ($a_{long}$, $a_{lat}$), yaw rate (Φ), wheel speeds (*v*), and front-axle steering angle (δ) at a 10 ms sampling time (100 Hz). Since all signals arrive within the same packet, alignment is performed automatically, ensuring that all sensor readings are synchronized without requiring additional processing. This guarantees that the data remain consistent across all measured parameters, facilitating accurate analysis and neural network training. The recorded measurements were then converted into csv files using custom Python 3.11. scripts for further processing.

The vehicle speed ranged from standstill to approximately 50 km/h, focusing on the forward trajectory without safety or comfort function interventions (ESC, adaptive cruise control (ACC), anti-lock braking system (ABS)).

Data recording occurred intermittently, with drivers stopping for at least 10 s every 5–10 min to capture a variety of driving conditions. The data segments between these stops are referred to as *data chunks*. Standstill periods were identified when the wheel speed sensor signal remained constant at 0.11 km/h. These standstill intervals, limited to 0.05 s, were excluded from the study as they served only to separate the data chunks.

To allow the examination of various driving styles, three scenarios were defined and performed: *calm driver*, *aggressive driver*, and *city driver*. Data for the first two scenarios were collected on the test track and the latter within Veszprém, Hungary. These scenarios reflect everyday driving habits and were thus included in the datasets. After the data cleaning phase, the retained durations for the *calm driver*, *aggressive driver*, and *city driver* scenarios were 3.23 h, 2.60 h, and 1.72 h, respectively. A fundamental statistical analysis of the sensor data collected during these scenarios is presented in Table 2, Table 3 and Table 4.

The primary distinctions among the driving scenarios are evident in the maximum and minimum values of the longitudinal and lateral accelerations and yaw rate. Specifically, the *aggressive driver* scenario exhibits higher forces impacting the vehicle. This trend extends to the yaw rate, where the maximum, minimum, and quartile values (Q1, Q2, Q3) associated with the *aggressive driver* scenario surpass those of the other scenarios significantly. Consequently, the dynamic behavior of the vehicle is notably more intense in the *aggressive driver* scenario, aligning with the characterization of an aggressive driving style. In this driving scenario, both the speed gradients and steering angles were increased.

In our research, we conducted the experiments on these three datasets separately, modeling the case where potential users collect a non-diverse dataset to train the network. Additionally, we compiled a mixed dataset from the three datasets, containing an equal proportion of data from each of the three driving scenarios. We will refer to this fourth dataset as *combined driver* and, in terms of the data volume, it contained a total of 7.55 h of data.

In addition to the *baseline data*, *supplementary data* were collected for the sensitivity analysis of the proposed LSTM model, following the same data collection methodology described in this section. More detailed information about the supplementary data can be found in Section 4.2.2.

To support further studies, all raw data collected are freely available for researchers on Figshare [34].

#### 4.1.2. Data Preprocessing

The input data were normalized within the range [−1,1] using expert scaling techniques. Under standard driving conditions, the acceleration sensor output typically falls within ±1 g, justifying its normalization within this range. The wheel speed was normalized from standstill to 70 km/h. Considering that the steering angle of the front axle does not exceed ±40°, this limit was used for normalization. The yaw rate was constrained to ±75°/s, a boundary that aligns with our empirical observations and practical experience.

To assess the applicability of the LSTM model, the dataset was divided into training, validation, and test sets while preserving the ratios of the different driving scenarios (calm, aggressive, city) across all subsets. A combined sampling approach was employed: stratified sampling was used to maintain the proportion of each driving scenario in the datasets, ensuring that the distribution remained consistent. Within each driving scenario, random sampling was applied to select individual data chunks. Approximately 75% of the total data was allocated for training and 25% for testing, and, within the training set, 10% was randomly selected for validation. In the following sections, these datasets will be referred to as the *training*, *validation*, and *test* data.

### 4.2. Methodology of Analyses

The practical applicability of the neural network model was assessed from four key perspectives. First, the necessary data volume for effective training was determined. Next, the impact of the tire pressure and the number of passengers on the model performance was evaluated. Lastly, the applicability of the model to entirely different vehicles was explored.

#### 4.2.1. Methodology to Determine the Required Amount of Data to Train the Proposed Model

During the research, a critical question emerged regarding how many data users need to effectively train the model on their own data. To determine the required data volume for the training of a well-performing model, the following method was employed. A random starting point was selected ten times within each driving scenario subset from the *baseline training dataset*. Starting from each starting point, datasets of various durations (1, 2, 3, 5, 10, 15, 20, 25, …, 60 min) were extracted. These selected datasets were then used to train the *base LSTM network structure*. During the training processes, 10% of the training dataset was randomly selected for validation purposes.

#### 4.2.2. Methodology of Sensitivity Analyses

During the sensitivity analysis of the model, we evaluated the impact of varying tire pressures, the influence of different passenger loads, and the model’s applicability across various vehicle body types. To achieve this, additional data were collected specifically for each analysis, simulating the different conditions under investigation (e.g., reduced tire pressure or increased passenger number). These newly acquired data were then used as test sets for the previously trained and optimized base LSTM network.

The dynamic behavior of a vehicle is significantly impacted by the tire pressure of the vehicle. Our primary focus was not on the scenario of a continuously decreasing tire pressure but rather on assessing how the neural network-based vehicle model responded to an extreme scenario where the tire pressure was significantly lower than normal. The tire pressure of the vehicle used for data collection described in Section 4.1.1 was initially 2.3 bar on all wheels. To investigate the effect of the tire pressure on the model, this pressure was reduced to 1.5 bar for each wheel separately. The selection of 1.5 bar as the reduced tire pressure level was intentional, as it represented a significant deviation from the original 2.3 bar, making its effects perceptible to both the driver and passengers. Given that the tire pressure directly influences the vehicle dynamics by altering the grip, rolling resistance, and slip angles, it was hypothesized that such a reduction would impact the accuracy of the yaw rate predictions. To systematically evaluate this effect, the model’s performance was analyzed under both the nominal and reduced tire pressure conditions, assessing its ability to generalize beyond the training domain. For the evaluation, data were gathered for the *aggressive*, *calm*, and *city* driver scenarios with reduced tire pressures. The aim was to evaluate the performance of the model, which was trained on data where all wheels had a tire pressure of 2.3 bar, in situations where the pressure of one wheel was extremely low.

The weight of the vehicle is also a crucial parameter influencing its dynamic behavior. In the data collection process described in Section 4.1.1, only the driver was present in the vehicle. To simulate the effect of an additional passenger, sandbags were used, with each sandbag weighing 20 kg. One passenger was simulated by placing five sandbags on a seat. Supplementary data were collected for all three driving scenarios by placing five sandbags separately on the front passenger seat, the rear passenger seat opposite the driver’s side, and the rear seat on the driver’s side. Additionally, a comprehensive weight configuration was examined, involving the placement of sandbags on all three mentioned passenger seats. The driver’s seat was, of course, occupied during all tests. The objective was to assess the performance of the model trained with data collected with only the driver present, in scenarios where additional passengers were also in the vehicle.

Finally, this research evaluated the performance of the model by applying it to different vehicle types. For this purpose, three other vehicles of varying sizes and drivetrains were utilized. The *Vehicle Original*, as referenced in Section 4.1.1, was a mid-size SUV. The types of vehicles used to collect supplementary data were as follows. *Vehicle A* represented a significantly smaller hatchback, while *Vehicle B* was another SUV, slightly larger than the *Vehicle Original*. *Vehicle C* was a substantial 7-seater SUV. It is important to note that both *Vehicle Original* and *Vehicle A* were equipped exclusively with internal combustion engines, whereas *Vehicle B* and *Vehicle C* were hybrids, featuring both internal combustion engines and electric drivetrains. Data were collected for each vehicle and for all three driving scenarios. The objective was to evaluate how a model trained on a combustion-engine mid-size SUV performed when applied to other vehicle types and sizes with different drivetrains.

#### 4.2.3. Evaluation Methodology

The evaluation of the results was carried out in each case by comparing them to the performance of the LSTM network trained on the *baseline data* (see Section 4.1). During the analysis, the deviations of the following error metrics were monitored: R-squared error ($R^{2}$), mean absolute error (MAE), mean squared error (MSE), and root mean squared error (RMSE) [1,35]. The exact calculations are presented in Equations (1)–(4).

(1) $$\Delta R_{i,j}^{2}=R_{i,j}^{2}-R_{i,\mathrm{base}}^{2}$$

(2) $$\Delta MAE_{i,j}=MAE_{i,j}-MAE_{i,\mathrm{base}}$$

(3) $$\Delta MSE_{i,j}=MSE_{i,j}-MSE_{i,\mathrm{base}}$$

(4) $$\Delta RMSE_{i}=RMSE_{i,j}-RMSE_{i,\mathrm{base}}$$

where *i* denotes the distinct driving scenario, including the *aggressive*, *calm*, *city*, and *combined* driving scenarios, and *j* represents the various analyses, including the volume of data utilized, tire pressure, additional passengers, and vehicle type. The notation *base* refers to the LSTM network trained on the *baseline datasets*.

This study was conducted on a high-performance workstation equipped with an Intel Xeon W-2265 CPU, an Nvidia A4000 GPU (16 GB VRAM), and 64 GB of RAM. The system ran Ubuntu 22.04, providing a stable and efficient environment for deep learning computations. TensorFlow 2.14 was used as the primary machine learning framework, leveraging GPU acceleration for training and testing. The hardware was selected to ensure the efficient processing of large datasets and real-time yaw rate prediction. This setup enabled the reliable execution of the experiments while maintaining computational efficiency.

## 5. Results

This section is organized into three main subsections. The first subsection presents the results after training and testing the LSTM network with the *baseline data*. As mentioned earlier, these results serve as reference points for comparisons. The subsequent subsection discusses the determination of the optimal amount of data required to train a well-performing network. The final subsection focuses on sensitivity analyses, examining the impact of different factors such as the tire pressure, number of passengers, and overall vehicle configuration.

### 5.1. Base LSTM Network

Training and testing the developed LSTM network structure using the *baseline dataset* was crucial in establishing a performance benchmark, allowing us to assess how well the model performed under standard conditions.

**Figure 2:**
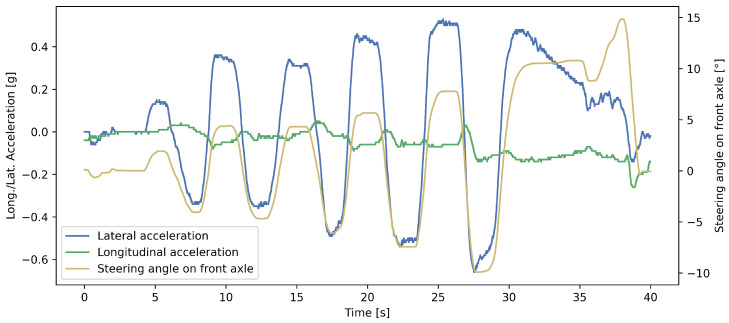
The longitudinal and lateral acceleration, along with the steering angle of the front axle, for a 40-s-long period selected from the test dataset.

**Figure 3:**
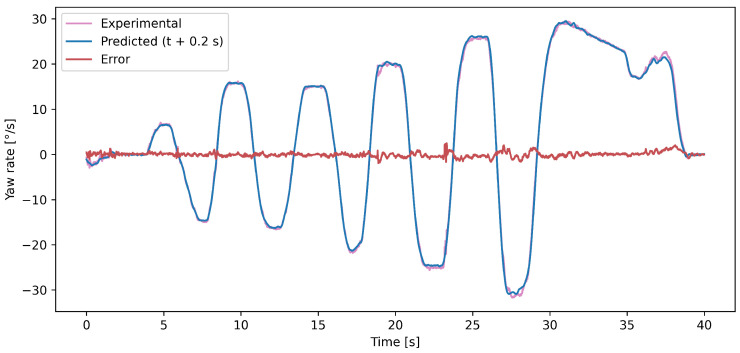
Forecast and observed yaw rate values for the period presented in Figure 2. The error was calculated as the signed difference between the experimentally derived and computationally predicted yaw rates.

Figure 2 illustrates the steering angle of the front axle, as well as the lateral and longitudinal acceleration, during a 40 s varied slalom maneuver selected from the test set. The vehicle, starting at an average speed of 40 km/h, eventually reached a standstill. The comparison between the forecast and measured yaw rate values over this interval is shown in Figure 3, highlighting the accuracy of the proposed LSTM model’s 0.2 s forecast. The mathematical relationship between these signals depends on the vehicle model used. Simplified models, such as the bicycle model, rely on only a few parameters, making them computationally efficient but less precise for complex dynamics. More advanced models, such as the four-wheel vehicle model, incorporate slip angles and load transfer, improving the accuracy at the cost of increased complexity. Unlike these physics-based approaches, our LSTM model learns the underlying relationships directly from the data, capturing both linear and nonlinear dependencies without requiring explicit equations. Neural network models excel in this regard: they achieve highly precise predictions without needing vehicle-dependent parameters as input, making them ideal for real-time vehicle control applications.

Table 5 presents the detailed performance metrics of the developed network across the three different driving scenarios and the combined one. Optimal fits were observed for both the training and validation sets, as expected, demonstrating their homogeneity. In the test dataset, the $R^{2}$ values were high for all driving scenarios, with the best results seen in the *calm driver* scenario ($R^{2}=0.9989$) and the lowest value ($R^{2}=0.9938$) observed in the *city driver* scenario. Regarding the model’s mean absolute error, the values ranged between 0.268 and 0.375 for the test set, but, when considering the training, validation, and test errors together, the MAE value did not exceed 0.470 in any scenario. Based on the calculated metrics for the test set, it can be concluded that the model’s output is suitable for use in safety-critical applications, as its error consistently remained within the mandatory threshold of 3 deg/s MAE, which is required to meet the highest safety standard (ASIL D) [36].

### 5.2. Determination of the Required Amount of Data to Train the Proposed Model

One possible step in implementing the proposed model involves adapting the LSTM structure to a specific vehicle type by training it with data collected from that vehicle. Therefore, two important questions arise regarding the number and type of data to be collected from the target vehicle to properly train the neural model and the impact of the size of the training dataset on the performance of the proposed LSTM network. To answer these questions, the *baseline dataset* was used as a training dataset and the research method introduced in Section 4.2.1 was applied.

**Figure 4:**
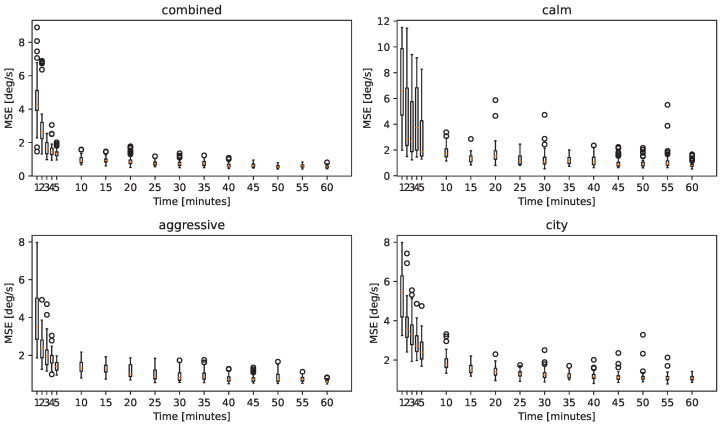
Box plot representation showing the relationship between the amount of data used to train the neural network and the accuracy of the fine-tuned network in different driving scenarios. Whiskers stretch from the edges of the box to the most distant data points that fall within 1.5 times the inter-quartile range (IQR).

The results are presented in Figure 4, showing the relationship between the predictive capability of the neural network and the volume of data used to train the network for different driving scenarios. The figures present the mean squared errors of the network on the test datasets. As shown, the best performance is achieved by using a dataset containing various driving styles (*combined dataset*); however, maneuvers characterized by strong dynamics, such as those of an *aggressive driver*, should be prioritized during data collection. In general, satisfactory results can be achieved by collecting 10–15 min of driving data, beyond which further improvements become difficult to attain. For comfort-related functions, this amount of data is sufficient to train an effective and practical neural network for vehicle dynamics predictions. However, in the context of safety-critical systems, the prevailing strategy is to utilize the maximum amount of data possible to ensure the highest level of accuracy and reliability.

### 5.3. Sensitivity Analyses

During the sensitivity analyses, several evaluations were conducted. In Table 6, the results regarding the impact of the reduced tire pressure are detailed. The most significant effect of the reduced tire pressure was observed in the *aggressive driver* scenario. From a tire dynamics perspective, the front wheels experience the highest impact due to their role as the steered axle. We must also note that the weight distribution for SUV vehicles is slightly larger on the front axle than the rear axle. In terms of the MAE, the maximum deviation is less than 0.5 deg/s (the ΔMAE for reduced tire pressure in the front left wheel is 0.4179).

In Table 7, the impact of the number and positions of passengers on the prediction of the vehicle’s yaw rate is presented for various passenger configurations (with the driver’s seat always occupied). In most cases, the most significant effects were observed in the *aggressive driver* scenario, and the lowest deviation in the error metrics was observed in the *calm driver* scenario. The smallest changes compared to the original metrics occurred when the driver and passenger were neither on the same side nor in the same row, followed by situations where the driver and passenger were either on the same side or in the same row. The largest deviation was observed when all seats, except the middle rear one, were unoccupied. Even in this case, the maximum MAE remained below 0.5 deg/s (0.4215 deg/s). In other cases, when only one additional person was seated in the car, the maximum MAE deviation did not exceed 0.13 deg/s.

In the final phase of the research, we investigated whether a model trained on a specific vehicle type could be applied to other types of vehicles. In other words, we examined how much the model’s error differed when applied to a different vehicle compared to the error measured on the vehicle used for training. The results are summarized in Table 8. Except for *Vehicle A*, the most significant impact is observed in the *aggressive driver* scenario, followed by the *calm driver* scenario. From a vehicle perspective, the largest differences are noted for the largest vehicle, *Vehicle C*, followed by *Vehicle B* and *Vehicle A*. The smallest effect is found for the combustion engine vehicle, *Vehicle A*, while a much larger difference is observed for the hybrid vehicle, *Vehicle B*, despite its size being close to that of the original vehicle. The purely electric *Vehicle C* also shows a significant impact. This table suggests that, rather than the overall weight of the vehicle, the weight distribution is much more important and should be considered when applying a model trained on different types of vehicles. To obtain more accurate conclusions, it is essential to analyze a greater number of vehicle types. Additionally, it should be noted that, even in these experiments, the MAE deviation from the reference values was less than 0.5 deg/s.

Overall, the sensitivity analyses show that the MAE error of the developed model, when considering both the original model error and the deviations in each scenario, consistently remained within the mandatory threshold of 3 deg/s MAE [36] in all investigated scenarios. This indicates that a well-trained model is suitable for real-time yaw rate prediction, as it effectively accounts for internal factors such as reduced tire pressure and variations in the number and positions of passengers, and it could also be applied for various types of vehicles.

## 6. Discussion

This study aimed to evaluate the applicability of the previously proposed LSTM model [1] in predicting the future yaw rate values of vehicles. The research focused on determining the prediction performance of the model, the determination of the required data to train the model, and a sensitivity analysis to assess the impact of the tire pressure, the number and positions of passengers, and the overall vehicle body dynamics.

The accuracy of the enhanced model cannot be directly compared to that of other models in the literature, as, to the best of our knowledge, no published models predict future yaw rate values. Therefore, we compare our results with published studies that estimated or computed the current yaw rate values based on data from other sensor signals. Since models published in the literature typically provide the RMSE values for model evaluation, we also use this metric for comparison purposes.

The EH∞KF filter proposed by Zhang et al. [27] uses 20 s of simulated data for a double-lane change. In their article, an RMSE of 1.378 deg/s was reported for the current yaw rate values. This RMSE is applicable only to the present yaw rate values, and not future ones, and relies on several vehicle-specific parameters, making implementation more complex.

The neural network model of Hermansdorfer et al. [22] was tested on three driving scenarios, and the authors reported an RMSE of 1.604 deg/s for the Monteblanco dataset. The model has a high resource demand (150 GRU units), which complicates its use in embedded systems. Additionally, the dataset used lacked information on the wheel speed, preventing direct model comparisons, and only provided current yaw rate values and not future predictions.

In the article on the “set membership” method [23], the authors used 211 s of experimental data and 5000 simulated data points from a single-track model. Their M2 model achieved the lowest yaw rate RMSE of 1.089 deg/s, demonstrating the highest accuracy among the models.

Comparing these results shows that the RMSE measure of our model (RMSE is 0.458, 0.366, 0.537, and 0.481 for *combined*, *calm*, *aggressive*, and *city datasets*, respectively) is even lower than the lowest values published in the literature (1.089). Although a direct comparison with published models is limited due to their focus on estimating the current yaw rate values rather than predicting future ones, our enhanced model still outperforms them in terms of overall performance and achieves superior results. Furthermore, it does so with significantly lower resource consumption, further highlighting its efficiency and effectiveness in future yaw rate prediction.

The tire pressure is a fundamental factor in vehicle stability and handling, directly influencing the interaction between the tires and the road surface. The sensitivity analysis demonstrated that deviations in the tire pressure, particularly in the front wheels, introduce changes in the yaw rate predictions. However, the LSTM model proves to be robust enough to compensate for these variations under typical operating conditions. While extreme deviations in the tire pressure can have an effect, modern vehicles are equipped with TPMSs that alert drivers before the pressure reaches critical levels. Therefore, the model remains highly applicable in real-world scenarios where the tire pressure is maintained within standard limits.

The passenger load distribution is another important factor in yaw rate prediction. This study found that asymmetric loading, such as when passengers are seated primarily on one side, results in a shift in the vehicle’s center of mass, leading to minor variations in the yaw rate. However, the impact remains minimal under normal conditions. Only in extreme cases, such as when the vehicle is fully occupied, with passengers seated in an unbalanced manner, does the yaw rate prediction slightly deviate. Even in these scenarios, the model maintains reliable performance, demonstrating that it is not overly sensitive to minor load distribution changes.

The vehicle body dynamics, particularly the difference between SUVs and electric vehicles, further highlight the strength of the proposed model. While SUVs tend to have a higher center of mass and more pronounced lateral weight shifts during cornering, electric vehicles benefit from lower battery placement, resulting in a more stable yaw response. Despite these differences, the LSTM model consistently delivers accurate predictions across both vehicle types. While fine-tuning could further optimize the predictions for specific architectures, the model already exhibits a high level of generalizability without requiring extensive modifications. This is an important strength, as it indicates that the model is not dependent on a specific vehicle type and can be applied across a broad range of platforms.

The amount of data available for the training of the model is another key factor in its accuracy. This study demonstrates that, for non-safety-critical applications, approximately 10–15 min of driving data are sufficient to achieve reliable predictions. For safety-critical scenarios, the use of a larger dataset ensures even greater precision, but the model already achieves high accuracy with reasonable amounts of data. The concern that more data could lead to computational inefficiencies is largely mitigated by the optimized architecture of the LSTM model, which remains efficient even when trained on larger datasets. The results of the sensitivity analysis showed that major fluctuations in the parameters of the vehicle do not significantly impact the prediction performance, reinforcing the practicality of deploying the model in real-world applications. This confirms that the LSTM network not only provides accurate predictions under controlled conditions but also maintains its efficiency under varying real-life constraints.

## 7. Summary

This study examined the performance of an improved LSTM model designed to predict vehicle yaw rates up to 0.2 s into the future, based on previous research. The key objectives included evaluating the predictive accuracy of the model, determining the amount of data required for effective training, and analyzing the sensitivity of the yaw rate predictions to variables such as the tire pressure, passenger load, and overall vehicle dynamics.

The model was trained using experimental data from various driving scenarios, including *aggressive*, *calm*, and *city driving*, with more than 7.5 h of data. In addition, a sensitivity analysis was conducted using 10 h of experimental data to evaluate the impact of different factors on the accuracy of the prediction.

The results indicate that this LSTM-based neural network model is capable of accurately forecasting the yaw rate under a wide range of conditions. The MAE of the model in all investigated cases remained below 0.5 deg/s, which is significantly lower than the 3 deg/s threshold defined by the ASIL D safety standard [36]. Therefore, our findings suggest that such neural network-based vehicle dynamics models have the potential to be applied in both safety-critical and non-safety-critical applications, improving vehicles’ performance and safety across various driving environments.

Although the results of our research are particularly promising, it is important to mention, as a limitation of the study, that the experimental data used to train, validate, and test the model were collected under various conditions, but the vehicle speeds during data collection ranged between 0 and 50 km/h. Therefore, our findings are only applicable to this speed range.

Currently, the real-world application of the developed LSTM-based yaw rate prediction model inside a test vehicle is ongoing. The model is being integrated into an embedded system to assess its real-time performance and validate its suitability for implementation in production vehicles. Parallel to this practical application, the necessary safety-related documentation is being created to ensure compliance with industry standards and regulations. The documentation process includes a detailed risk assessment, the verification of functional safety requirements, and validation procedures in accordance with ISO 26262 guidelines [36]. This step is essential in demonstrating the reliability and robustness of the proposed model, paving the way for its potential use in safety-critical automotive applications. The integration phase will also provide further insights into potential improvements in computational efficiency and the feasibility of deploying such neural network-based vehicle dynamics models in commercial automotive systems.

## Figures and Tables

**Table 1:** The parameter space used for hyperparameter tuning. The bold values refer to the results of the process. The sigmoid function refers to $sigmoid\left(x\right)=1/\left(1+e^{\left(-x\right)}\right)$ and the hard sigmoid function is defined as 0 if x<−2.5, 1 if x>2.5, and 0.2x+0.5 if −2.5≤x≤2.5 [33].

Hyperparameter	Values
Number of hidden layers	**1**, 2, 3, 4
Number of LSTM neurons per layer	1, 2, 3, 4, **5**, 6, 7, 8, 9, 10
Batch size	32, 64, 128, 256, **512**, 1024, 2048
Learning rate	0.0001, **0.001**, 0.01
Mode of weight initialization	**normal random**, uniform random
LSTM activation function	**tanh**, sigmoid, hard sigmoid
Recurrent activation function	tanh, **sigmoid**, hard sigmoid
Dropout rate	**0**, 0.1, 0.2, 0.3
n_look_back	10, 20, **30**, 40, 50

**Table 2:** Statistical properties of the *calm driver* data.

	$a_{\mathrm{long}}$ [g]	$a_{\mathrm{lat}}$ [g]	Φ [°/s]	δ [°]	$v_{\mathrm{FL}}$ [km/h]	$v_{\mathrm{FR}}$ [km/h]	$v_{\mathrm{RR}}$ [km/h]	$v_{\mathrm{RL}}$ [km/h]
Mean	−0.02	0.01	0.491	1.49	36.53	36.66	36.33	36.45
Std	0.05	0.21	3.879	10.93	9.86	9.73	9.86	9.72
Min	−0.49	−0.73	−33.149	−36.63	0.11	0.11	0.11	0.11
Q1 (25%)	−0.03	−0.14	−1.915	−6.78	31.25	31.55	31.05	31.31
Q2 (50%)	−0.01	0.00	0.049	0.17	37.12	37.14	36.93	36.93
Q3 (75%)	0.00	0.17	2.321	8.50	42.66	42.79	42.45	42.57
Max	0.35	0.62	30.953	42.91	62.52	63.00	62.35	62.90

**Table 3:** Statistical properties of the *aggressive driver* data.

	$a_{\mathrm{long}}$ [g]	$a_{\mathrm{lat}}$ [g]	Φ [°/s]	δ [°]	$v_{\mathrm{FL}}$ [km/h]	$v_{\mathrm{FR}}$ [km/h]	$v_{\mathrm{RR}}$ [km/h]	$v_{\mathrm{RL}}$ [km/h]
Mean	−0.03	0.01	1.376	0.42	36.16	36.28	35.88	36.00
Std	0.13	0.32	15.935	5.34	8.88	8.81	8.91	8.84
Min	−0.85	−0.95	−64.562	−33.31	0.11	0.11	0.11	0.11
Q1 (25%)	−0.08	−0.20	−8.689	−2.56	32.11	32.28	31.81	31.98
Q2 (50%)	−0.02	0.00	0.372	0.11	37.35	37.41	37.08	37.14
Q3 (75%)	0.04	0.23	11.397	3.22	42.09	42.07	41.86	41.86
Max	0.50	0.95	68.011	33.25	58.64	58.07	58.51	57.60

**Table 4:** Statistical properties of the *city driver* data.

	$a_{\mathrm{long}}$ [g]	$a_{\mathrm{lat}}$ [g]	Φ [°/s]	δ [°]	$v_{\mathrm{FL}}$ [km/h]	$v_{\mathrm{FR}}$ [km/h]	$v_{\mathrm{RR}}$ [km/h]	$v_{\mathrm{RL}}$ [km/h]
Mean	−0.01	0.01	0.020	−0.04	30.27	30.21	30.16	30.10
Std	0.06	0.07	5.888	4.10	12.68	12.65	12.68	12.66
Min	−0.37	−0.46	−35.513	−31.11	0.11	0.11	0.11	0.11
Q1 (25%)	−0.02	−0.01	−0.841	−0.36	21.16	21.28	21.04	21.14
Q2 (50%)	−0.01	0.00	−0.046	−0.02	33.03	32.95	32.93	32.86
Q3 (75%)	0.02	0.02	0.549	0.19	40.39	40.30	40.28	40.21
Max	0.30	0.46	35.255	33.19	56.40	56.26	56.67	56.63

**Table 5:** Error metrics of the proposed model on the *baseline dataset*.

	Combined	Calm	Aggressive	City
Training set				
Length (min)	328.4	139.2	112.0	77.2
$R^{2}$ (1)	0.9980	0.9989	0.9977	0.9955
MAE (deg/s)	0.338	0.262	0.470	0.285
MSE ((deg/s)^2^)	0.294	0.125	0.598	0.155
RMSE (deg/s)	0.542	0.354	0.773	0.394
Validation set				
Length (min)	36.6	15.5	12.5	8.6
$R^{2}$ (1)	0.9980	0.9989	0.9977	0.9955
MAE (deg/s)	0.338	0.262	0.469	0.286
MSE ((deg/s)^2^)	0.291	0.126	0.590	0.157
RMSE (deg/s)	0.540	0.354	0.768	0.397
Test set				
Length (min)	92.1	39.0	31.6	21.5
$R^{2}$ (1)	0.9984	0.9989	0.9985	0.9938
MAE (deg/s)	0.316	0.268	0.375	0.316
MSE ((deg/s)^2^)	0.210	0.134	0.288	0.231
RMSE (deg/s)	0.458	0.366	0.537	0.481

**Table 6:** The impact of reduced tire pressure (1.5 bar) on the model performance relative to the performance of the *base model* (2.3 bar on each tire).

	Combined	Calm	Aggressive	City
Reduced $p_{FL}$				
Length (min)	50.0	10.2	9.3	30.5
Δ $R^{2}$ (1)	−0.0042	0.0002	−0.0054	0.0005
ΔMAE (deg/s)	0.1231	−0.0064	0.4179	−0.0104
ΔMSE ((deg/s)^2^)	0.3946	−0.0244	2.0328	−0.0648
ΔRMSE (deg/s)	0.3195	−0.0350	0.9868	−0.0729
Reduced $p_{FR}$				
Length (min)	50.9	10.7	10.1	30.1
Δ $R^{2}$ (1)	−0.0013	0.0004	−0.0020	−0.0011
ΔMAE (deg/s)	0.0559	0.0063	0.3238	−0.0113
ΔMSE ((deg/s)^2^)	0.1851	−0.0030	1.1919	−0.0829
ΔRMSE (deg/s)	0.1705	−0.0042	0.6799	−0.0957
Reduced $p_{RL}$				
Length (min)	50.6	10.4	10.0	30.2
Δ $R^{2}$ (1)	−0.0003	0.0004	−0.0002	−0.0005
ΔMAE (deg/s)	0.0614	0.0257	0.1532	0.0394
ΔMSE ((deg/s)^2^)	0.1003	−0.0016	0.5172	−0.0305
ΔRMSE (deg/s)	0.0989	−0.0021	0.3607	−0.0328
Reduced $p_{RR}$				
Length (min)	55.0	11.1	11.0	32.9
Δ $R^{2}$ (1)	0.0001	0.0005	0.0005	0.0003
ΔMAE (deg/s)	0.0431	−0.0128	0.1432	0.0257
ΔMSE ((deg/s)^2^)	0.0362	−0.0311	0.2596	−0.0348
ΔRMSE (deg/s)	0.0380	−0.0452	0.2034	−0.0377

**Table 7:** The impact of the number and positions of passengers on the model performance relative to the performance of the *base model*.

	Combined	Calm	Aggressive	City
Front passenger seat occupied				
Length (min)	51.4	10.3	9.9	31.2
Δ $R^{2}$ (1)	−0.0015	−0.0007	−0.0003	−0.0033
ΔMAE (deg/s)	0.1091	0.0258	0.1173	0.1321
ΔMSE ((deg/s)^2^)	0.1368	0.1203	0.3126	0.0674
ΔRMSE (deg/s)	0.1308	0.1382	0.2383	0.0656
Rear passenger seat (passenger side) occupied				
Length (min)	50.9	10.7	10.1	30.1
Δ $R^{2}$ (1)	−0.0006	0.0003	0.0003	−0.0021
ΔMAE (deg/s)	0.0516	0.0142	0.1027	0.0448
ΔMSE ((deg/s)^2^)	0.1120	−0.0151	0.2418	0.0928
ΔRMSE (deg/s)	0.1093	−0.0212	0.1912	0.0884
Rear passenger seat (driver side) occupied				
Length (min)	50.2	10.7	10.4	29.1
Δ $R^{2}$ (1)	−0.0011	0.0004	−0.0008	−0.0008
ΔMAE (deg/s)	0.0572	0.0177	0.1253	0.0437
ΔMSE ((deg/s)^2^)	0.1532	−0.0079	0.5165	0.0611
ΔRMSE (deg/s)	0.1445	−0.0110	0.3603	0.0598
All seats are occupied, except the rear middle one				
Length (min)	52.5	10.4	10.6	31.5
Δ $R^{2}$ (1)	−0.0025	−0.0006	−0.0029	−0.0030
ΔMAE (deg/s)	0.1533	0.1137	0.4215	0.0734
ΔMSE ((deg/s)^2^)	0.3768	0.2400	1.4528	0.0418
ΔRMSE (deg/s)	0.3080	0.2454	0.7828	0.0416

**Table 8:** The impact of the entire vehicle body on the model performance relative to the performance of the *base model*.

	Combined	Calm	Aggressive	City
Vehicle A				
Length (min)	77.3	20.8	21.7	34.8
Δ $R^{2}$ (1)	−0.0008	−0.0005	−0.0003	−0.0033
ΔMAE (deg/s)	0.0718	0.0954	0.0436	0.0668
ΔMSE ((deg/s)^2^)	0.2103	0.2123	0.3756	0.0809
ΔRMSE (deg/s)	0.1902	0.2223	0.2780	0.0778
Vehicle B				
Length (min)	75.1	21.3	22.0	31.8
Δ $R^{2}$ (1)	−0.0010	−0.0008	−0.0010	−0.0015
ΔMAE (deg/s)	0.1792	0.1960	0.3545	0.0375
ΔMSE ((deg/s)^2^)	0.3614	0.2845	0.9115	0.0066
ΔRMSE (deg/s)	0.2979	0.2808	0.5586	0.0068
Vehicle C				
Length (min)	57.0	11.3	10.2	35.5
Δ $R^{2}$ (1)	−0.0045	−0.0016	−0.0057	−0.0005
ΔMAE (deg/s)	0.1247	0.0749	0.4396	0.0486
ΔMSE ((deg/s)^2^)	0.3215	0.1148	1.5206	0.0247
ΔRMSE (deg/s)	0.2710	0.1327	0.8082	0.0250

## Data Availability

The original data presented in the study are openly available via FigShare at https://doi.org/10.6084/m9.figshare.28078274.v1 (accessed on 22 February 2025) [34], according to MDPI Research Data Policies.

## Abbreviations

The following abbreviations are used in this manuscript:

ABS	Anti-Lock Braking System
ACC	Adaptive Cruise Control
ANN	Artificial Neural Network
ASIL	Automotive Safety Integrity Level
CSV	Comma-Separated Values
EH∞KF	Extended H-Infinity Kalman Filter
EKF	Extended Kalman Filter
ESC	Electronic Stability Control
FL	Front Left (Wheel)
FR	Front Right (Wheel)
GRU	Gated Recurrent Unit
IMU	Inertial Measurement Unit
IQR	Inter-Quartile Range
ISO	International Organization for Standardization
LSTM	Long Short-Term Memory
MAE	Mean Absolute Error
MSE	Mean Squared Error
OEM	Original Equipment Manufacturer
Q1	First Quartile (25th percentile)
Q2	Second Quartile (50th percentile, median)
Q3	Third Quartile (75th percentile)
RAM	Random Access Memory
RL	Rear Left (Wheel)
RMSE	Root Mean Squared Error
RR	Rear Right (Wheel)
SUV	Sport Utility Vehicle
TPMS	Tire Pressure Monitoring System
VRAM	Video Random Access Memory
